# Supplementary material for: Evidence of a causal and modifiable relationship between kidney function and circulating trimethylamine N-oxide
Source: Nat Commun. 2023 Sep 20;14:5843. doi: 10.1038/s41467-023-39824-4 (PMC10511707; doi:10.1038/s41467-023-39824-4)
Supplement: Supplementary file 1 — Supplementary Information [file 41467_2023_39824_MOESM1_ESM.pdf]

Supplementary Information For

Evidence of a causal and modifiable relationship between kidney function and circulating trimethylamine N-oxide.

Andrikopoulos P, Aron-Wisnewsky J, et al.

Supplemental Figures

Supplemental Figure 1. TMAO is associated with worse cardiometabolic profiles in BMIS.

Supplemental Figure 2. Correcting urinary metabolites for urinary creatinine does not improve TMAO predictions.

Supplemental Figure 3. LASSO linear-regression models explain less circulating TMAO variance in BMIS than boosted trees models.

Supplemental Figure 4. TMAO is the measured metabolite most strongly associated with eGFR in BMIS.

Supplemental Figure 5. CAG01909, associates with higher circulating TMAO in BMIS (N=834) individuals.

Supplemental Figure 6. Boosted decision tree algorithms predict circulating TMAO in IHD MetaCardis participants.

Supplemental Figure 7. *R. timonensis* inversely associates with circulating TMAO whilst BMIS-identified CAG01909 does not in overt disease.

Supplemental Figure 8. TMAO activates ERK1/2 via a Ca^{2+}-sensitive pathway in human renal fibroblasts.

Supplemental Figure 9. TMAO or choline diet exacerbates renal fibrotic injury *in vivo*.

Supplemental Figure 10. Propensity-score matching of MetaCardis patients with T2D.

Supplemental Tables

Supplemental Table 1. Characteristics of MetaCardis sub-cohorts (BMIS, T2D, IHD).

Supplemental Table 2. BMIS TMAO cluster characteristics.

Supplemental Table 3. Model hyperparameters and N numbers for each variable group in BMIS.

Supplemental Table 4. Model hyperparameters and N numbers for each variable group in T2D.

Supplemental Table 5. Model hyperparameters and N numbers for each variable group in IHD.

Supplemental Table 6. Characteristics of MetaCardis participants prescribed GLP-1RAs and matched controls.

Supplemental Figure 1

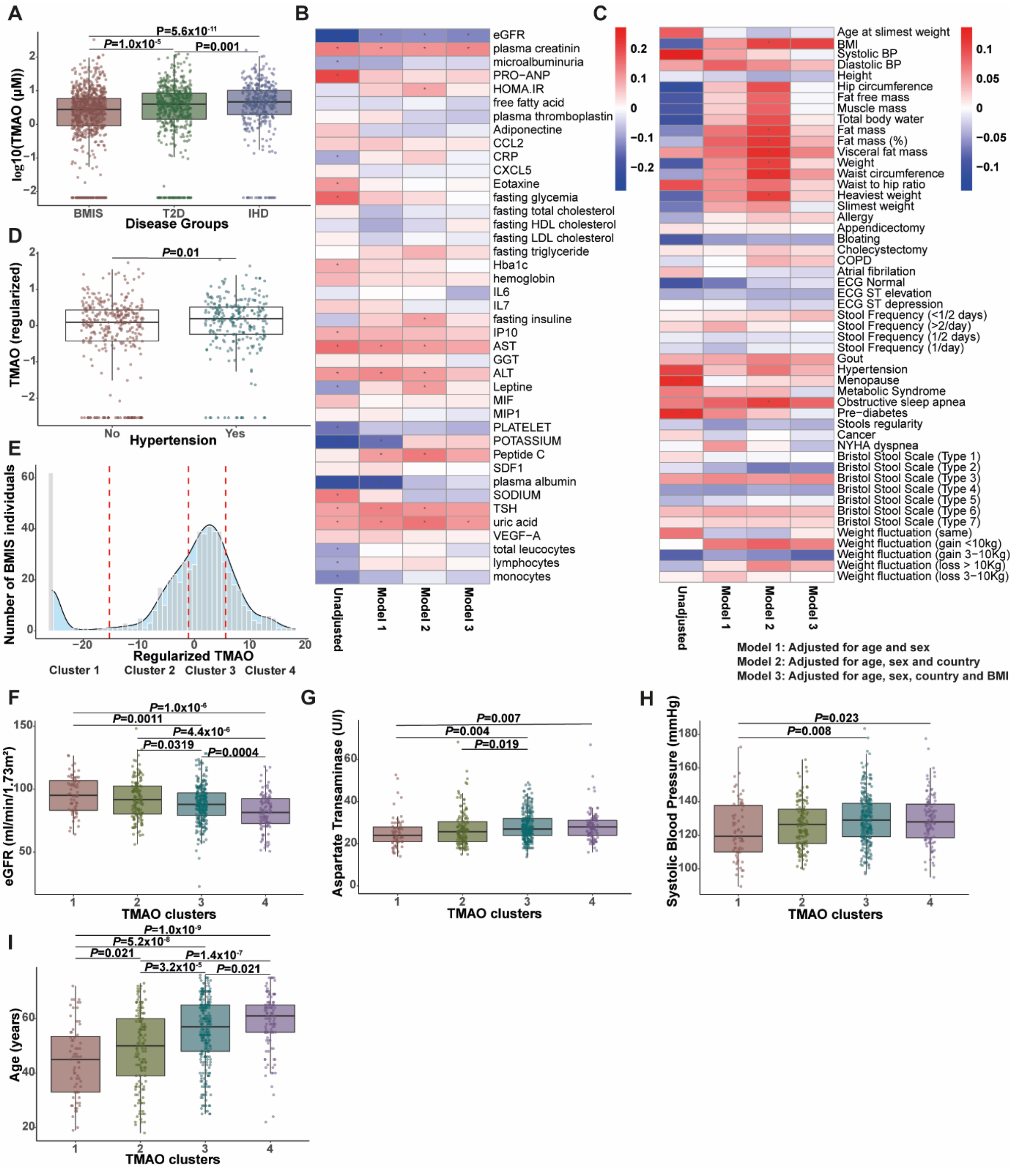

Supplemental Figure 1. TMAO is associated with worse cardiometabolic profiles in study participants with metabolic syndrome-related risk factors or pathologies but not over cardiometabolic disease (BMIS subgroup N=581). (A) Boxplot illustrating differences in fasting circulating TMAO levels in the three MetaCardis patient groups (Suppl.Table.1 for sub-cohort characteristics). P determined by two-sided Mann-Whitney U test. (**B**) Spearman correlations between circulating TMAO levels and bioclinical variables unadjusted, adjusted for age and sex (Model 1), age sex and country of recruitment (Model 2) or age, sex country of recruitment and BMI (Model 3), *pFDR<0.1. (**C**) Spearman correlations between plasma TMAO and clinical variables, corrected as in

(A). (**D**) Comparison of circulating TMAO levels between BMIS participants classed as hypertensives, P determined by two-sided Mann-Whitney U test. (**E**) Density plot illustrating the distribution of regularized circulating TMAO levels in BMIS (N=582). The population was split into four TMAO clusters using the k-means algorithm. Dashed red lines denote the cut-off values for this distribution. Between group comparisons of selected variables for BMIS participants split into clusters according to their circulating TMAO levels for eGFR (**F**; mL/min/1.73m^2), aspartate transaminase (**G**; U/l), systolic blood pressure (**H**; mmHg) and age at the time of recruitment (**I**, years). P values were determined with pairwise two-sided Mann-Whitney U tests corrected for multiple comparisons with the Benjamini-Hochberg method. For (A), (D) and (F-I) center lines denote medians, box limits indicate the 25th and 75th percentiles, whiskers extend to the minimal and maximal values. Source data are provided as a Source Data file.

Supplemental Figure 2

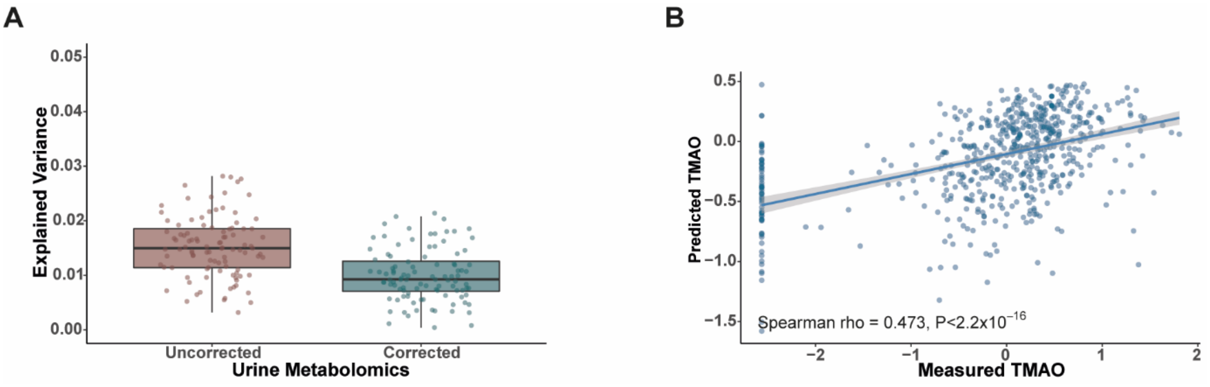

Supplemental Figure 2. Correcting urinary metabolites for urinary creatinine does not improve TMAO predictions and predicted circulating TMAO by the full model in BMIS significantly correlates with measured TMAO. (**A**) Coefficients of determination (Explained Variance) of predicted circulating TMAO levels determined by xgboost algorithms after 5-fold cross-validation in the left-out group (Suppl.Table.3 for N numbers and optimized xgboost parameters), trained with input urinary metabolite absolute levels computed by the IVDr algorithm from ^{1}H-NMR spectra, excluding methylamines (TMA and dimethylamine) and corrected by urinary creatinine (also computed with the IVDr pipeline; Corrected) *versus* TMAO Explained Variance computed from the absolute levels of the same metabolites uncorrected for creatinine (Uncorrected). Center lines denote medians, box limits indicate the 25th and 75th percentiles, whiskers extend to the minimal and maximal values. (**B**) Predicted (averaged after 100 iterations; y-axis) regularized plasma TMAO of BMIS participants by the BMIS-trained full-model versus actual measured regularized TMAO values (x-axis) for BMIS individuals (N=582); insert Spearman rho and *P*-value of predicted *versus* measured TMAO values. The shaded area indicates 95% confidence interval. Source data are provided as a Source Data file.

Supplemental Figure 3

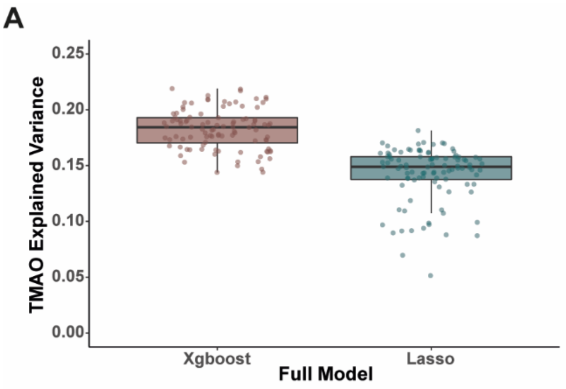

Supplemental Figure 3. LASSO linear-regression models explain less circulating TMAO variance in BMIS (N=582) than boosted trees models. Explained Variance of predicted circulating TMAO levels determined by LASSO linear regression models in BMIS (N=582) after 5-fold cross-validation in the left-out group for 100 iterations. Explained variance from boosted trees models (Xgboost) from BMIS full model computed in Figure 2A are also included for comparison. Center lines denote medians, box limits indicate the 25th and 75th percentiles, whiskers extend to the minimal and maximal values. Source data are provided as a Source Data file.

Supplemental Figure 4

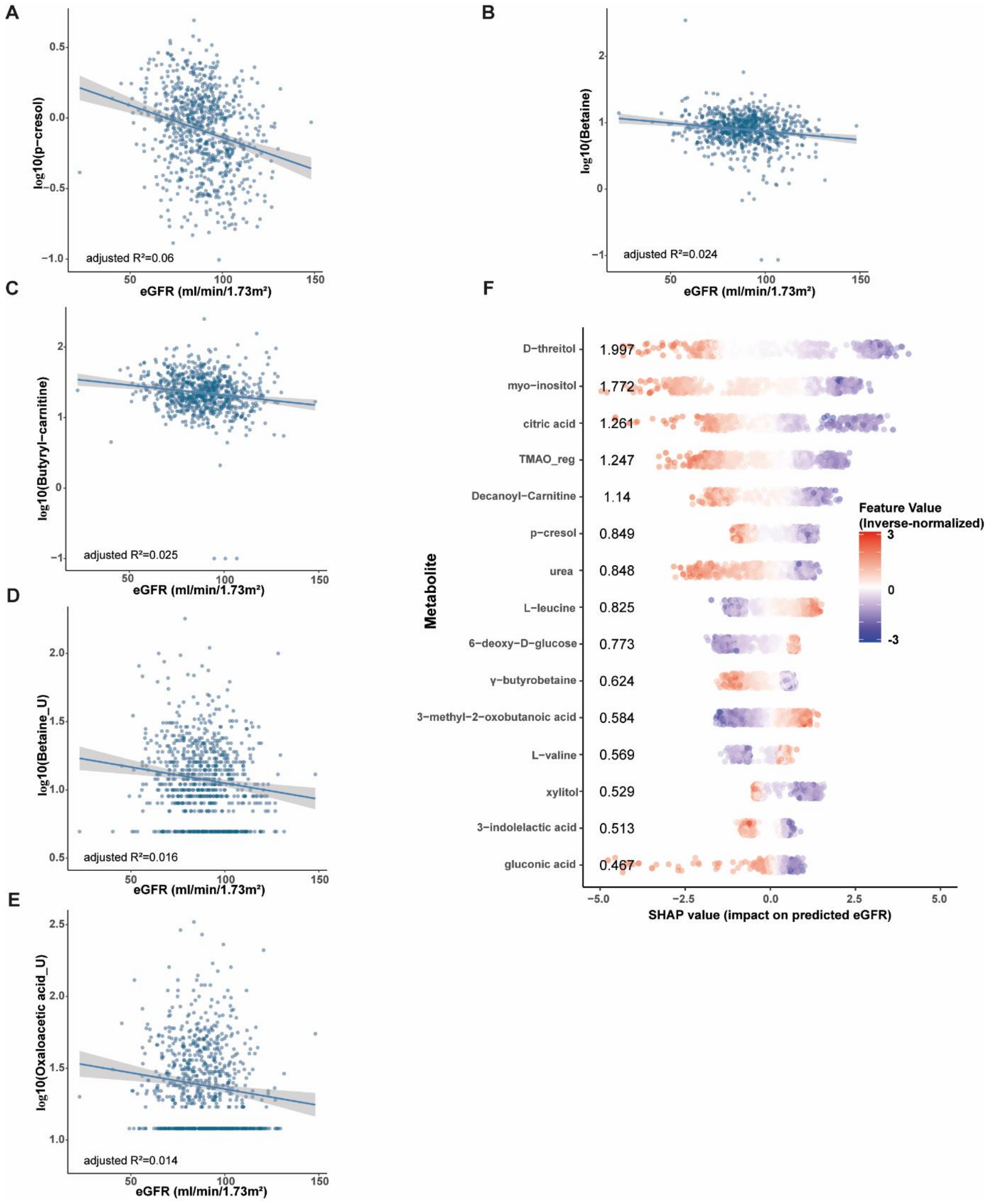

Supplemental Figure 4. TMAO is the measured metabolite most strongly associated with eGFR in BMIS (N=767). Linear-regression-based scatterplot showing correlation between metabolites most strongly predictive of TMAO (Figure 2C; serum p-cresol (**A**), serum betaine (**B**), serum butyryl-carnitine (**C**), urinary betaine (**D**), urinary oxaloacetic acid (**E**)) as dependent variables and estimated Glomerular Filtration Rate (eGFR, ml/min/1.73m^2) as independent variable. Insert, explained variance (R^2). For A-E the shaded area indicates 95% confidence intervals. (**F**) Swarm plots of SHAP values (impact on eGFR model predictions) for each BMIS MetaCardis participant with available serum metabolomics (N=767);

represented by individual dots, for the top 20 metabolites contributing to eGFR predictions, computed from xgboost algorithms trained on serum metabolomics. Numbers denote mean absolute SHAP values from all BMIS participants (in descending order) next to their corresponding metabolite. Dots are colored by the inverse-normalized value of their corresponding metabolite. See Suppl.Table.3 for N numbers and optimized xgboost parameters. Source data are provided as a Source Data file.

Supplemental Figure 5

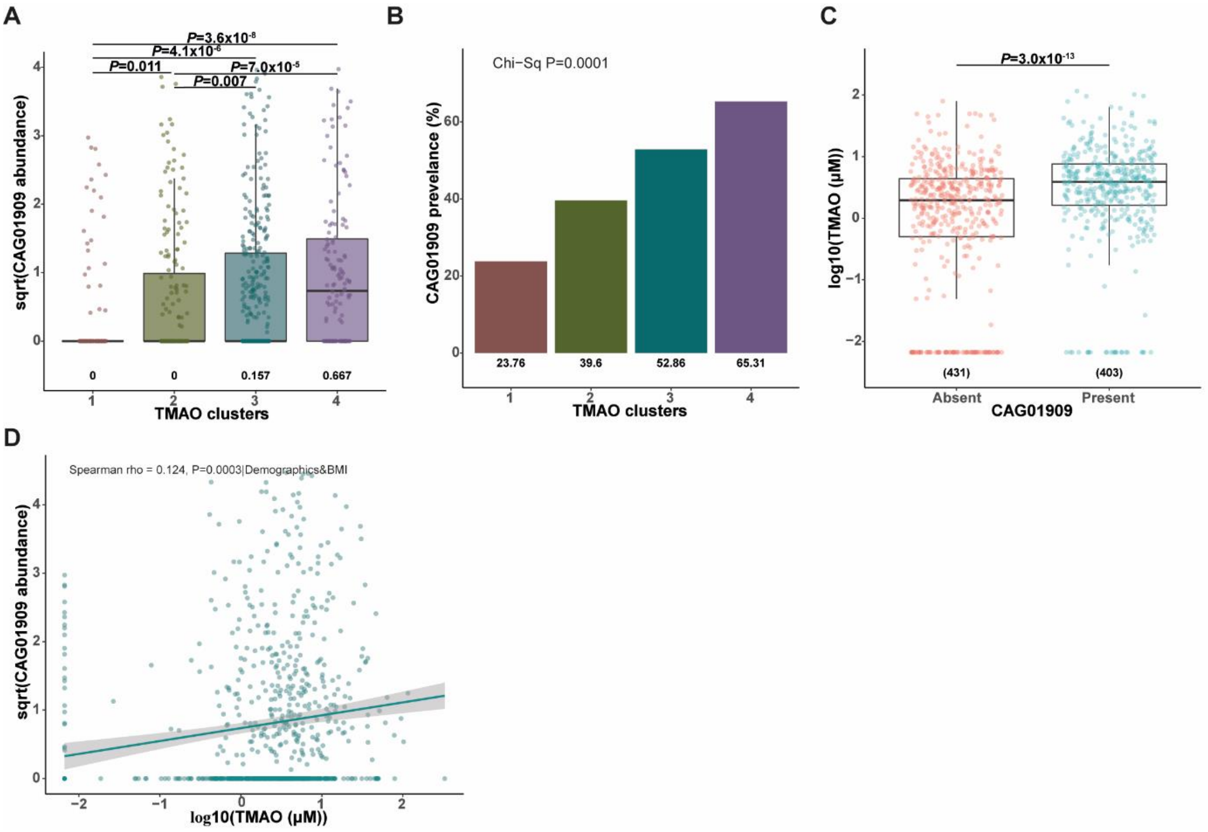

Supplemental Figure 5. CAG01909, associates with higher circulating TMAO in BMIS (N=834) individuals. Comparisons of normalized to bacterial load expression levels (square-root transformed) of (**A**) CAG01909, an unknown bacterium in BMIS participants divided into clusters according to circulating TMAO levels with the k-means algorithm. Median abundance level for each cluster is shown below the corresponding box plot, all pairwise comparisons performed with two-sided Mann-Whitney U tests (corrected for multiple testing with the Benjamini-Hochberg method). (**B**) Prevalence ((%); in parentheses under corresponding boxplots) of *CAG01909* in BMIS participants (N=834) split into TMAO clusters as in (A). *P* value determined by two-sided Chi-square test. (**C**) BMIS participants were split into those where CAG01909 is present in their gut microbiota (N=401) and those where it is absent (N=433) and circulating log-transformed TMAO levels between the two groups were compared (two-sided Mann-Whitney U test). (**D**) Linear-regression-based scatterplot showing correlation between normalized by bacterial load CAG01909 abundance levels (square-root transformed for visualization purposes) and TMAO ($\log_{10}$-transformed). Adjusted (age, sex, country of recruitment and BMI) Spearman rho=0.123 and pFDR=0.032 for CAG01909 (see source data for Figure 3C). The shaded area indicates 95% confidence interval. For (A) and (C) center lines denote medians, box limits indicate the 25th and 75th percentiles, whiskers extend to the minimal and maximal values. Source data are provided as a Source Data file.

Supplemental Figure 6

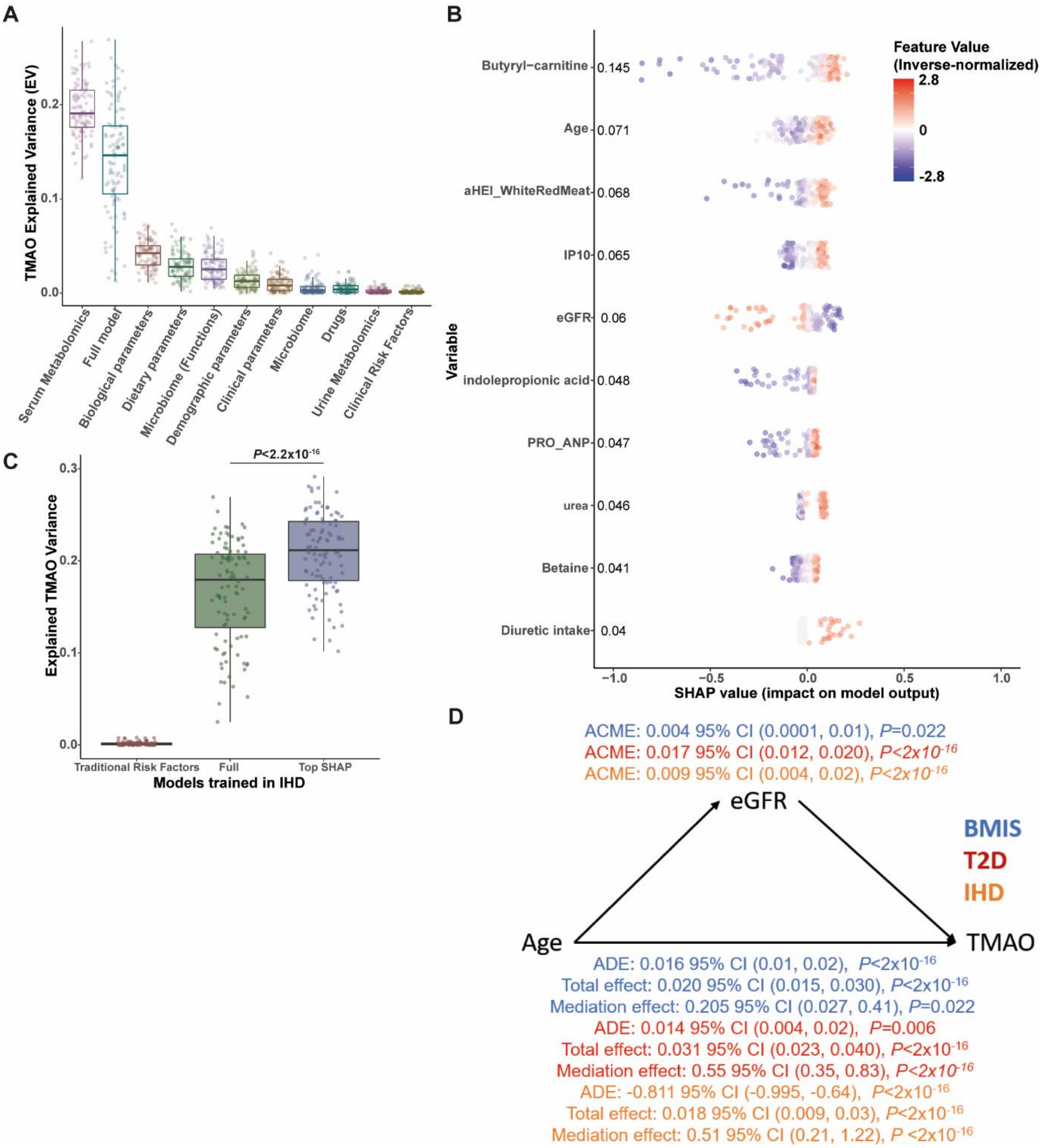

Supplemental Figure 6. Boosted decision tree algorithms predict circulating TMAO in IHD MetaCardis participants. (A) Explained Variance (EV) of predicted serum TMAO levels determined by boosted decision trees (methods), trained exclusively on variables from each feature category (Supplemental Data 1 for a list of variables included in each group), or the full model (containing all variables), after 100 iterations (Suppl.Table.5 for N numbers and optimized xgboost parameters per variable group) in IHD MetaCardis individuals. (**B**) Swarm plots of SHAP values (impact on model outcome; *x-axis*) for each IHD MetaCardis participant with complete phenotypic data (N=221), for all variables contributing to model predictions more than 4% of regularized TMAO standard deviation, computed from xgboost algorithms trained on each feature category. Numbers denote mean absolute SHAP values from all IHD participants (in descending order) next to their corresponding variable. Dots, representing IHD individuals, are colored by the inverse-normalized value of their corresponding

variable. (**C**) Boxplots depicting Explained Variance (EV; R^2) of circulating TMAO in IHD individuals computed by algorithms trained on clinical risk factors[29], the full model containing all variables or all the variables contributing more than 4% of regularized TMAO standard deviation to IHD model predictions, as determined by SHAP analysis, after 100 iterations. *P*-value was determined by a two-sided Mann-Whitney U test, corrected for multiple testing with the Benjamini-Hochberg method (**D**) Mediation analysis computing the direct effect of eGFR on TMAO increase with age in BMIS (blue), T2D (red) or IHD (orange) MetaCardis participants. ADE: Average direct effect (of Age on TMAO); ACME: average mediation effect (of eGFR on TMAO); Total effect: (cumulative effect of age and eGFR on TMAO (ADE + ACME)); Mediation effect: (% of the effect of age on circulating TMAO attributed to eGFR). For (A) and (E) center lines denote medians, box limits indicate the 25th and 75th percentiles, whiskers extend to the minimal and maximal values. Source data are provided as a Source Data file.

Supplemental Figure 7

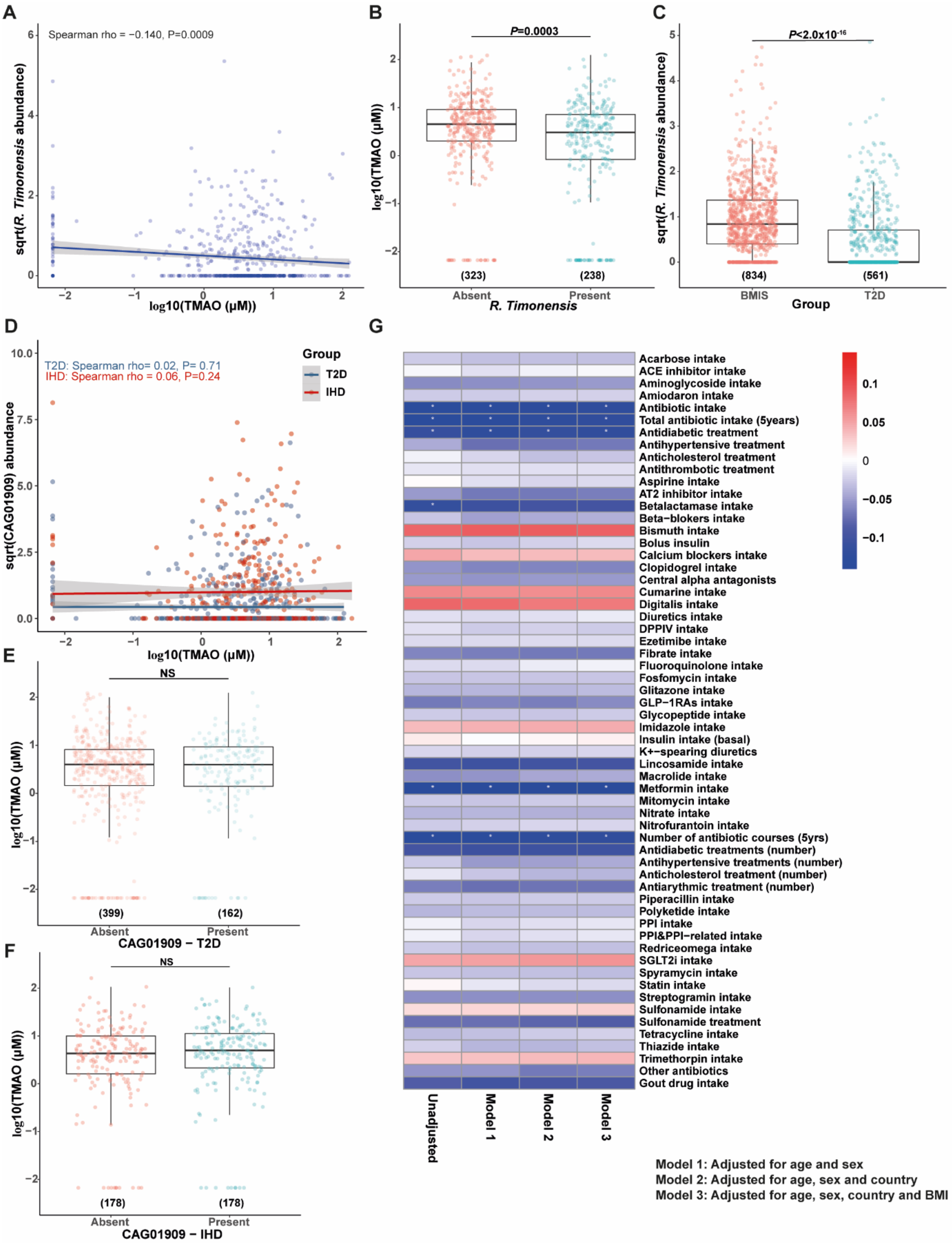

Supplemental Figure 7. In MetaCardis T2D participants *R. timonensis* inversely associates with circulating TMAO whilst BMIS-identified CAG01909 does not in overt disease. (**A**) Linear-regression-based scatterplot showing associations between normalized by bacterial load *R. timonensis* abundance levels (square-root transformed for visualization purposes) and TMAO (log_{10}-transformed). Unadjusted spearman rho=-0.140 and P=0.009. The shaded area indicates 95% confidence interval (**B**)

Comparison of log-transformed circulating TMAO between T2D individuals with detectable (present) and undetectable (absent) fecal *R. timonensis* (two-sided Mann-Whitney test). Number of individuals in each group shown in parenthesis. (**C**) Boxplot illustrating differences in *R. timonensis* fecal abundance in the BMIS and T2D MetaCardis patient groups (group sizes in parentheses), *P* determined by two-sided Mann-Whitney U test. (**D**) Linear-regression-based scatterplot showing correlation between normalized by bacterial load CAG01909 expression levels (square-root transformed for visualization purposes) and TMAO ($\log_{10}$-transformed) in T2D and IHD MetaCardis subjects. The shaded area indicates 95% confidence interval. Comparison of log-transformed circulating TMAO between T2D (**E**) and IHD (**F**) MetaCardis subjects with detectable (present) or undetectable (absent) fecal CAG01909 (two-sided Mann-Whitney test). Number of individuals in each group shown in parenthesis. (**G**) Heatmap illustrating Spearman correlations between bacterial load-normalized abundance of CAG01909 and intake of medication in T2D MetaCardis individuals (N=561) unadjusted, adjusted for age and sex (Model 1), age sex and country of recruitment (Model 2) or age, sex country of recruitment and BMI (Model 3), *pFDR<0.1. For (B-C) and (E-F) center lines denote medians, box limits indicate the 25th and 75th percentiles, whiskers extend to the minimal and maximal values. Source data are provided as a Source Data file.

Supplemental Figure 8

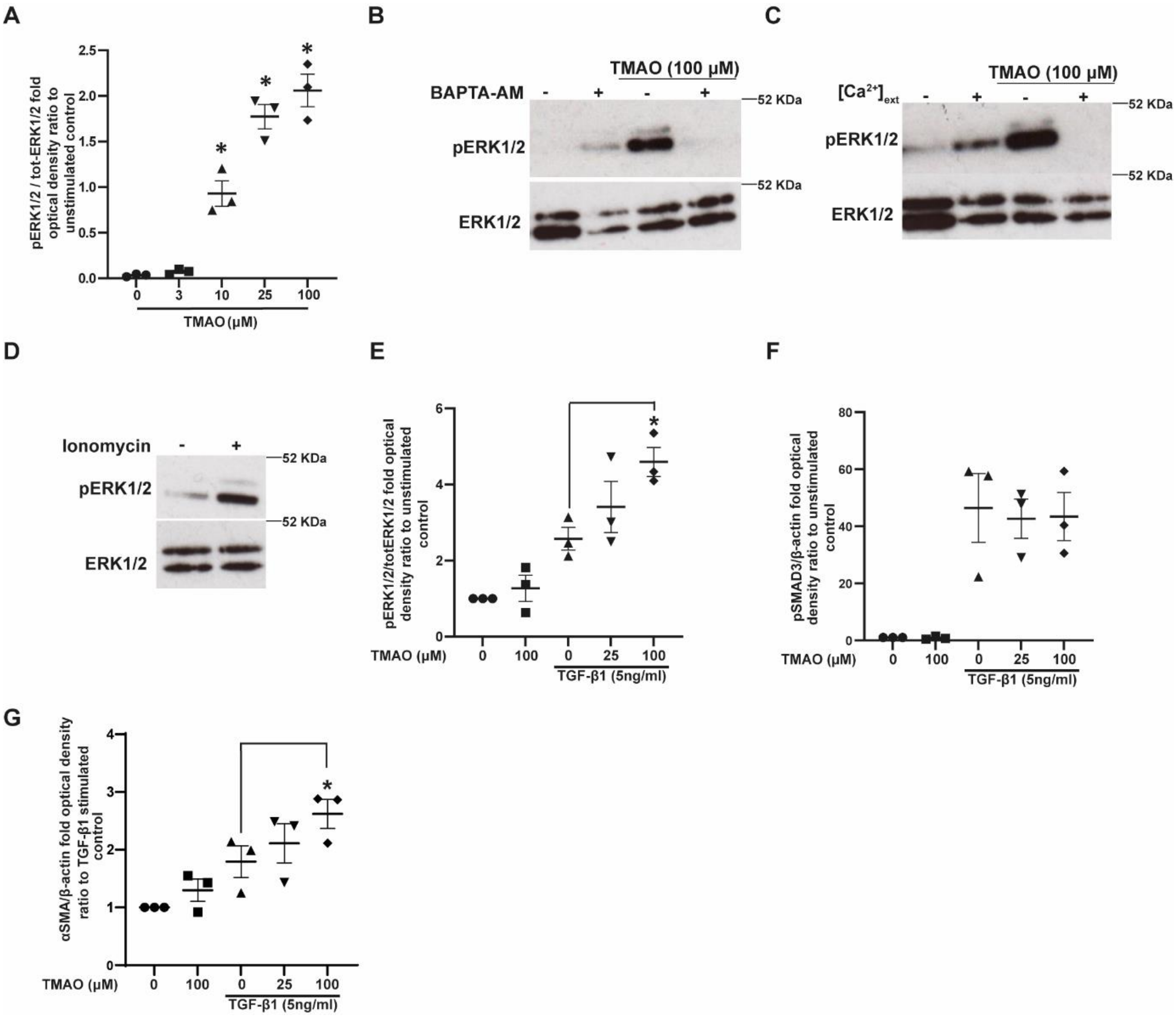

Supplemental Figure 8. TMAO activates ERK1/2 via a Ca^{2+}-sensitive pathway in human renal fibroblasts. (A) Optical density (OD) of pERK1/2 levels normalized against total ERK1/2 for experiments represented in Figure 5C. The normalized density of the control unstimulated samples was arbitrarily set to 1. *P<0.05 *versus* the unstimulated control, unpaired, one-tailed, Student t-test. (**B**) Serum-starved Human Renal Fibroblasts (HRFs) were loaded with the Ca^{2+} chelator BAPTA-AM (20μM; 20min) and then stimulated with TMAO (100μM) for 10min. ERK1/2 activation was probed by Western blot and membranes were stripped and re-probed for total ERK1/2, a representative image of N=3 independent experiments is shown. (**C**) HRFs were serum-starved in complete physiological medium for 45min, the medium was aspirated and cells were incubated for further 15min in physiological medium with Ca^{2+} omitted from the buffer. Subsequently, cells were stimulated with 100μM TMAO for 10min and ERK1/2 activation was probed as in (**B**). (**D**) Serum-starved HRFs were stimulated with the Ca^{2+} ionophore ionomycin (100μM) and phospho-ERK1/2 levels were probed as in (B). OD of pERK1/2 (**E**) or pSMAD3 (**F**) normalized by total ERK1/2 or β-actin respectively for experiments represented in Figure 5D. The normalized density of the control unstimulated samples was arbitrarily set to 1. *P<0.05 *versus* the TGF-β1 - stimulated control, unpaired, one-tailed, Student t-test. (**G**) OD of αSMA normalized by β-actin levels for experiments represented in Figure 5E. *P<0.05 versus the TGF-β1 - stimulated control, unpaired, one-tailed, Student t-test. For all N=3 independent experiments, error bars represent ±SEM. Source data are provided as a Source Data file.

Supplemental Figure 9

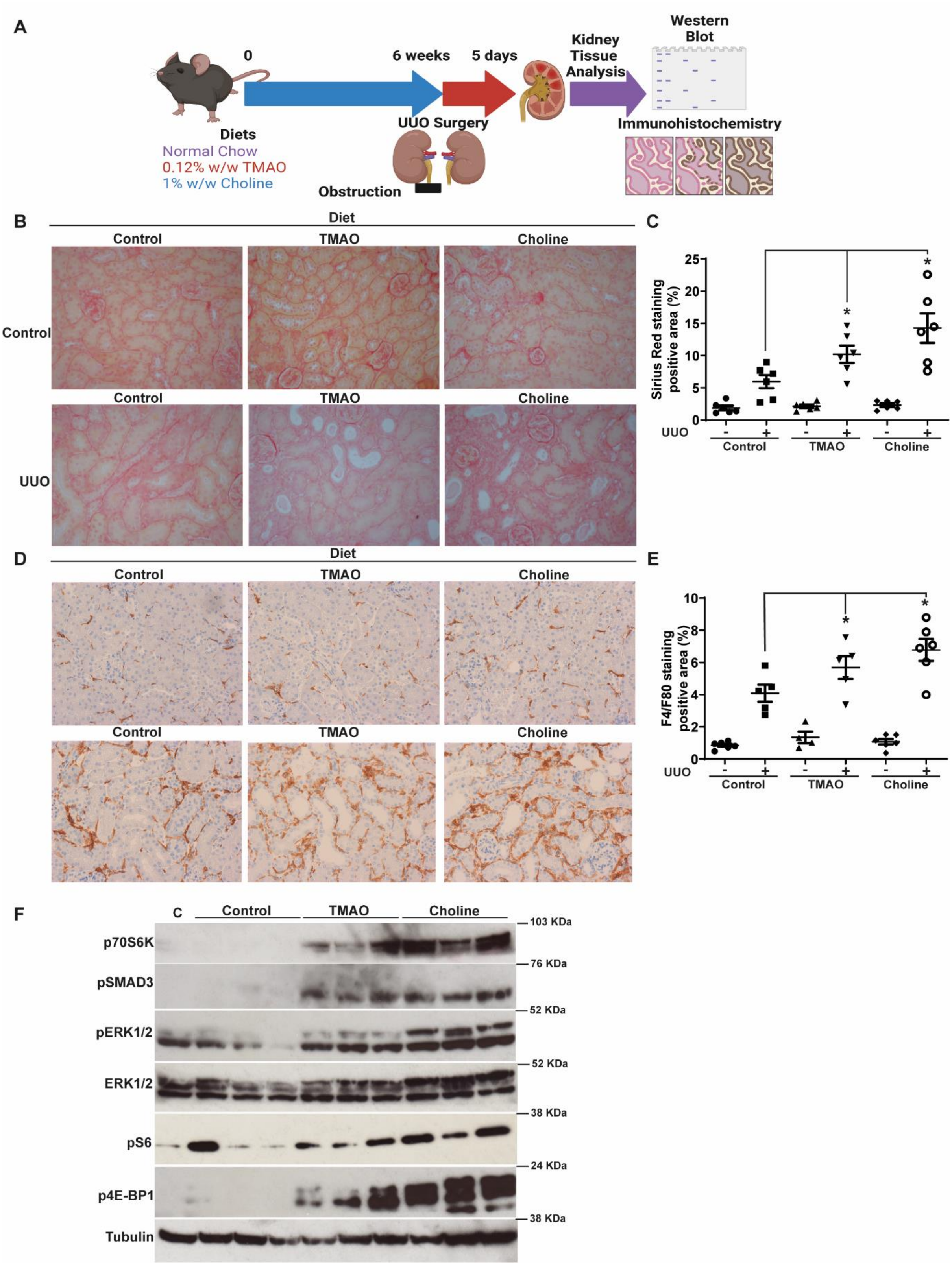

Supplemental Figure 9. TMAO or choline diet exacerbates collagen deposition, macrophage infiltration and activate pro-fibrotic signaling in kidneys of mice that underwent Unilateral Ureter Obstruction (UUO) surgery. (**A**) Outline of the animal experiments. Created with BioRender.com (**B**) Sirius red staining of kidney sections (20x magnification) from obstructed (UUO; 5days post-surgery)

or contralateral sham-operated (control) kidneys. Animals were fed normal chow (control), a diet containing 0.12% w/w TMAO (TMAO) or 1% choline w/w (Choline) for 6weeks prior to surgery, as indicated. N=6 per group. (**C**) Quantification of collagen deposition staining as (%) of positive Sirius red area/field of view averaged from 5 images per animal. *P<0.05 versus the Sirius red staining in the control UUO kidney, unpaired, one-tailed, Student t-test. (**D**) Immunostaining of kidney sections (20x magnification) from UUO or control kidneys as in (**B**) with the macrophage marker F4/F80. (**E**) Quantification of macrophage infiltration as % of F4/F80 staining/ field of view of images from (**D**); N=6 animals/group. *P<0.05 versus the F4/F80 staining in the control UUO kidney, unpaired, one-tailed, Student t-test. (**F**) Western blot for profibrotic signaling pathways activation from UUO kidneys from animals treated as in (**A**). C: contralateral kidney from a control-diet fed animal; TMAO: 0.12% w/w TMAO diet; Choline: 1% w/w Choline diet. Source data are provided as a Source Data file.

Supplemental Figure 10

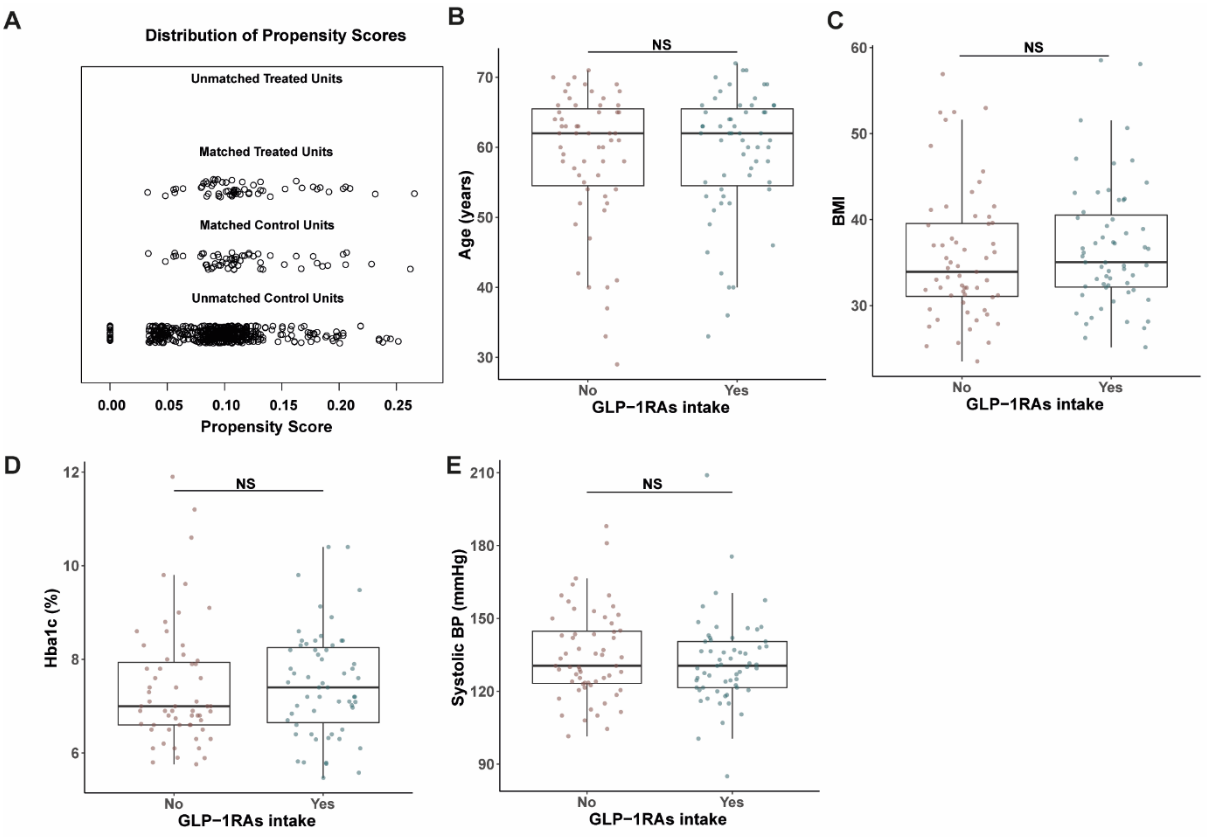

Supplemental Figure 10. Propensity-score matching of MetaCardis patients with T2D. (A) Propensity scores of MetaCardis participants with T2D taking GLP-1 Receptor agonists (GLP-1RAs); N=59) and matched controls (N=59) using nearest neighbors matching with age, sex, disease severity group and hypertension status as covariates (Suppl.Table.6). Comparisons of age (**B**), BMI (**C**), glycated hemoglobin (**D**) and systolic blood pressure (**E**), between MetaCardis patients with T2D diabetics prescribed GLP-1RAs (N=59) and matched controls (as in A; N=59). Two-sided Mann-Whitney U tests were used to determine statistical significance for B-E. NS: non-significant (*P*>0.05). For (B-E) center lines denote medians, box limits indicate the 25th and 75th percentiles, whiskers extend to the minimal and maximal values.

Supplemental Table 1.

	BMIS	T2D	IHD	P_BMIS_vs_T2D	P_BMIS_vs_IHD	P_T2D_vs_IHD
N	837	561	356			
Age (years)	55	62	63	**<2e-16**	**<2e-16**	**0.0018**
Male (%)	35.7	52.4	83.4	**6.20E-10**	**< 2e-16**	**< 2e-16**
Female (%)	64.3	47.7	16.6	**6.20E-10**	**< 2e-16**	**< 2e-16**
TMAO (µmol/l)	2.788	3.991	4.676	**0.000013**	**5.6E-11**	**0.0098**
eGFR (mL/min/1.73m^2)	88.94	85.63	84.7	**2.30E-05**	**6.20E-09**	**0.038**
BMI (kg/m^2)	30.71	32.23	27.73	**6.10E-06**	**1.20E-06**	**< 2e-16**
HbA1c (%)	5.58	6.89	5.8	**<2e-16**	**<2e-16**	**<2e-16**
HOMA.IR	2.065	4.277	2.583	**< 2e-16**	**2.90E-07**	**< 2e-16**
Fasting Triglycerides (mmol/l)	1.12	0.1505	1.186	**<2e-16**	0.077	**6.00E-13**
Fasting Total Cholesterol (mmol/l)	5.1	4.69	3.75	**8.00E-15**	**<2e-16**	**<2e-16**
Fasting HDL Cholesterol (mmol/l)	1.37	1.21	1.075	**9.20E-14**	**< 2e-16**	**6.20E-08**
Fasting LDL Cholesterol (mmol/l)	3.18	2.735	1.987	**<2e-16**	**<2e-16**	**<2e-16**
Systolic Blood Pressure (mmHg)	127.5	134	125.5	**6.90E-11**	0.056	**8.40E-12**
Diastolic Blood Pressure (mmHg)	73	75.5	70	**0.0019**	**6.10E-06**	**9.20E-11**
Hypertension (%)	46.3	77.7	92.6	**< 2e-16**	**< 2e-16**	**1.30E-07**
Type-2 Diabetes (%)	0	100	29.8	**<2e-16**	**<2e-16**	**<2e-16**
Germany (%)	29.7	39.6	0	**0.00014**	**< 2e-16**	**< 2e-16**
Denmark (%)	30.8	8	60.4	**<2e-16**	**<2e-16**	**<2e-16**
France (%)	39.5	52.4	39.6	**5.20E-06**	0.95364	**0.00031**
Current smoker (%)	11.9	12.7	15.8	0.69	0.27	0.43
Past smoker (%)	40.1	47.2	59.6	**0.0118**	**2.70E-08**	**0.0014**
Never smoked (%)	41.1	31.2	21.5	**0.00058**	**5.80E-09**	**0.00334**
Anti-hypertensive treatment (%)	33.2	67.4	90.2	**< 2e-16**	**< 2e-16**	**2.90E-15**
Anti-diabetic treatment (%)	0	85.6	19.7	**<2e-16**	**<2e-16**	**<2e-16**
Anti-cholesterol treatment (%)	13.3	43.7	90.7	**<2e-16**	**<2e-16**	**<2e-16**
Anti-thrombotic treatment (%)	1.2	1.4	33.4	0.71	<2e-16	<2e-16
Metformin intake (%)	0	73.1	16.6	**<2e-16**	**<2e-16**	**<2e-16**
AT2 Receptor Blocker intake (%)	13.6	31.6	23.9	**1.80E-15**	**2.80E-05**	**0.012**
ACE inhibitor intake (%)	9.1	26.4	36	**<2e-16**	**<2e-16**	**0.0021**
Statin intake (%)	11.9	39.9	89	**<2e-16**	**<2e-16**	**<2e-16**

Supplemental Table 1. Characteristics of the MetaCardis sub-cohorts. Patients were divided in sub-groups according to clinical characteristics. BMIS MetaCardis participants (N=837) diagnosed with metabolic syndrome or in the presence of risk factors thereoff but not overt Type-2 Diabetes (T2D) or ishaemic heart disease (IHD). T2D (N=561) MetaCardis participants diagnosed with T2D but not IHD. IHD (N=356) MetaCardis individuals with acute coronary syndrome, Chronic coronary artery disease or heart failure. Estimated Glomerular Filtration Rate (eGFR), Glycated Haemoglobin (HbA1c), Homeostatic Model Assessment for Insulin Resistance (HOMA.IR), Center of recruitment (Germany,

Denmark, France). All numeric values are median. Pairwise statistical significance was determined with two-sided Wilcoxon rank sum tests. *P<0.05.

Supplemental Table 2.

	Cluster 1	Cluster 2	Cluster 3	Cluster 4	P_Cl_1_vs_Cl_2	P_Cl_1_vs_Cl_3	P_Cl_1_vs_Cl_4	P_Cl_2_vs_Cl_3	P_Cl_2_vs_Cl_4	P_Cl_3_vs_Cl_4
N	67	142	268	105						
Age (years)	45	50	57	61	0.039	8.70E-08	1.00E-09	6.30E-05	2.90E-07	0.039
Male (%)	26.9	30.3	38.1	37.1	1	0.53	0.66	0.59	0.78	1
Female (%)	73.1	69.7	61.9	62.9	1	0.53	0.66	0.59	0.78	1
TMAO (µmol/l)	0.01	0.84	3.58	12	<2e-16	<2e-16	<2e-16	<2e-16	<2e-16	<2e-16
eGFR (mL/min/1.73m²)	95.1	91.6	87.9	81.5	0.1226	0.0022	1.00E-06	0.0531	7.40E-06	0.0008
BMI (kg/m²)	27.8	35.8	29.3	28.4	0.27	1	1	0.19	0.15	1
HbA1c (%)	5.43	5.54	5.58	5.5	0.497	0.047	0.241	0.812	1	1
HOMA.IR	1.86	2.09	2.02	1.96	0.93	1	1	1	1	1
Fasting Triglycerides (mmol/l)	0.844	1.23	1.12	1.01	0.7	0.15	0.86	0.7	0.86	0.18
Fasting Total Cholesterol (mmol/l)	4.9	5.01	5.29	5	0.006	0.008	0.152	0.38	0.15	0.38
Fasting HDL Cholesterol (mmol/l)	1.44	1.39	1.37	1.37	0.43	0.43	0.33	1	1	1
Fasting LDL Cholesterol (mmol/l)	2.9	3.27	3.3	3.1	0.229	0.048	0.834	0.834	0.834	0.329
Systolic Blood Pressure (mmHg)	120	126	129	128	0.27	0.009	0.038	0.137	0.27	0.859
Diastolic Blood Pressure (mmHg)	70	72.5	73	74.5	1	0.93	0.93	1	1	1
Hypertension (%)	23.9	47.9	44	50.5	0.005	0.011	0.003	0.912	0.913	0.786
Type-2 Diabetes (%)	0	0	0	0	-	-	-	-	-	-
Germany (%)	55.2	52.8	28.4	13.3	0.75	9.80E-05	2.50E-08	4.30E-06	1.10E-09	0.005
Denmark (%)	9	20.4	38.8	52.4	0.04	1.40E-05	4.30E-08	0.00047	8.50E-07	0.035
France (%)	35.8	26.8	32.8	34.3	1	1	1	1	1	1
Current smoker (%)	10.4	15.5	13.4	5.71	1	1	1	1	0.1	0.17
Past smoker (%)	31.3	37.3	39.9	54.3	0.802	0.591	0.2	0.802	0.041	0.048
Never smoked (%)	55.2	37.3	40.7	32.4	0.075	0.128	0.019	0.846	0.846	0.418
Anti-hypertensive treatment (%)	10.4	37.3	32.1	37.1	0.0004	0.0017	0.0006	0.862	0.978	0.86
Anti-diabetic treatment (%)	0	0	0	0	-	-	-	-	-	-
Anti-cholesterol treatment (%)	4.48	9.86	11.9	17.1	0.557	0.374	0.082	0.557	0.374	0.557
Anti-thrombotic treatment (%)	0	0.7	1.49	1.9	1	1	1	1	1	1
Metformin intake (%)	0	0	0	0	-	-	-	-	-	-
AT2 Receptor Blocker intake (%)	4.48	14.1	13.1	14.3	0.23	0.23	0.23	1	1	1
ACE inhibitor intake (%)	4.48	13.4	7.46	10.5	0.31	1	0.65	0.31	1	1
Statin intake (%)	2.99	9.86	10.4	16.2	0.329	0.281	0.044	0.852	0.379	0.379

Supplemental Table 2. Characteristics of BMIS (with metabolic syndrome or risk factors thereof but not overt Type-2-Diabetes of ischemic heart disease) MetaCardis subjects divided into clusters according to circulating TMAO. BMIS individuals with full phenotypic data (N=582) were split into clusters according to circulating TMAO levels using the k-means algorithm. Estimated Glomerular Filtration Rate (eGFR), Glycated Haemoglobin (HbA1c), Homeostatic Model Assessment for Insulin Resistance (HOMA.IR), Center of recruitment (Germany, Denmark, France). All numeric values are median. Pairwise statistical significance was determined with two-sided Wilcoxon rank sum tests. *P<0.05.

Supplemental Table 3.

Variable Group	N	eta	max_depth	min_child_weight	gamma	lamda	colsample_bytree	nrounds
Biological parameters	837	0.1	3	1	0	1	0.8	32
Clinical parameters	795	0.035	1	3	0.75	0	0.8	243
Demographic parameters	795	0.05	2	3	0.8	0.05	0.9	59
Drugs	837	0.025	2	7	0.75	0	0.8	180
Dietary parameters	763	0.1	2	1	0.5	0.8	0.8	26
Microbiome (Functions)	834	0.0125	2	1	0.2	0	0.8	172
Microbiome	834	0.01	9	11	0.1	0	0.9	148
Clinical Risk Factors	837	0.025	2	4	0	0	0.9	119
Serum Metabolomics	771	0.05	7	5	0.5	0.1	0.8	89
Urine Metabolomics	742	0.0025	3	6	1	0	0.8	1024
Full model	582	0.035	1	8	0.75	0	0.9	312
Top SHAP	582	0.01	3	1	1	0	0.9	333
Urine Metabolomics (corrected for creatinine)	742	0.05	5	2	6	0	0.9	57
eGFR	767	0.075	2	7	0.1	0	0.8	133

Supplemental Table 3. Xgboost algrorithm parameters per variable category in BMIS. For each of our 10 variable groups we optimized xgboost models using 5-fold cross-validation and two sequential hyperparameter grids searches (972 parameter combinations per feature group) using root-mean-square error (RMSE) to evaluate outcomes (methods). For each variable groups optimal xgboost parameters used to determine explained variance and SHAP values (methods) and numbers of BMIS MetaCardis subjects with available data (N) are appended.

Supplemental Table 4. Model hyperparameters and N numbers for each variable group in T2D.

Variable Group	N	eta	max_depth	min_child_weight	gamma	lamda	colsample_bytree	nrounds
Biological parameters	561	0.1	4	7	1	0.25	0.9	234
Clinical parameters	521	0.01	8	7	0.5	0	0.8	143
Demographic parameters	504	0.025	2	6	0	0	0.8	100
Drugs	561	0.025	2	6	0.2	0	0.8	99
Dietary parameters	477	0.025	4	6	0	0	0.8	63
Microbiome (Functions)	561	0.01	5	8	0.75	0	0.8	152
Microbiome	561	0.025	2	6	0	0	0.9	112
Clinical Risk Factors	561	0.01	2	2	0	0	0.9	304
Serum Metabolomics	532	0.05	8	8	0.5	0.5	0.8	92
Urine Metabolomics	515	0.01	2	5	0.5	0	0.8	218
Full model	387	0.015	1	8	0	0	0.8	263
Top SHAP	387	0.0075	1	7	0	0	0.9	1470

Supplemental Table 4. Xgboost algrorithm parameters per variable category in T2D. For each of our 10 variable groups we optimized xgboost models using 5-fold cross-validation and two sequential hyperparameter grids searches (972 parameter combinations per feature group) using root-mean-square error (RMSE) to evaluate outcomes (methods). For each variable groups optimal xgboost parameters used to determine explained variance and SHAP values (methods) and numbers of T2D MetaCardis subjects with available data (N) are appended.

Supplemental Table 5. Model hyperparameters and N numbers for each variable group in IHD.

Variable Group	N	eta	max_depth	min_child_weight	gamma	lamda	colsample_bytree	nrounds
Biological parameters	356	0.1	3	8	4	0	0.8	19
Clinical parameters	272	0.015	6	8	0.25	0	0.9	19
Demographic parameters	297	0.01	6	4	0.25	0	0.8	102
Drugs	356	0.0025	8	4	1	0	0.9	352
Dietary parameters	298	0.025	3	6	1.5	0	0.9	48
Microbiome (Functions)	356	0.05	2	8	0	0	0.8	21
Microbiome	356	0.01	9	3	0.25	0	0.9	14
Clinical Risk Factors	329	0.01	2	1	0	0	0.9	136
Serum Metabolomics	339	0.01	3	2	1.5	0.05	0.8	310
Urine Metabolomics	327	0.0125	2	8	0.5	0	0.8	143
Full model	221	0.0075	6	4	0.1	0.1	0.8	381

Supplemental Table 5. Xgboost algrorithm parameters per variable category in IHD. For each of our 10 variable groups we optimized xgboost models using 5-fold cross-validation and two sequential hyperparameter grids searches (972 parameter combinations per feature group) using root-mean-square error (RMSE) to evaluate outcomes (methods). For each variable groups optimal xgboost parameters used to determine explained variance and SHAP values (methods) and numbers of IHD MetaCardis subjects with available data (N) are appended.

Supplemental Table 6. Characteristics of MetaCardis participants prescribed GLP-1RAs and matched controls.

	Prescribed GLP-1RAs	Matched-Controls	P-Value
N	59	59	-
Age (years)	62	62	0.93
Male (%)	59.3	55.9	0.71
Female (%)	40.7	44.1	0.71
TMAO (µmol/l)	3	7.08	**0.022**
eGFR (mL/min/1.73m^2)	81.3	82.7	0.93
BMI (kg/m^2)	35	33.9	0.28
HbA1c (%)	7.4	7	0.3
HOMA.IR	7.02	4.64	**0.028**
Fasting Triglycerides (mmol/l)	1.89	1.52	0.096
Fasting Total Cholesterol (mmol/l)	4.18	4.44	0.098
Fasting HDL Cholesterol (mmol/l)	1.01	1.08	0.11
Fasting LDL Cholesterol (mmol/l)	2.22	2.66	0.11
Systolic Blood Pressure (mmHg)	130	130	0.51
Diastolic Blood Pressure (mmHg)	75	74.5	0.79
Hypertension (%)	89.8	89.8	1
Type-2 Diabetes (%)	100	100	-
Ischemic Heart Disease (%)	13.6	11.9	0.79
Germany (%)	30.5	13.6	**0.027**
Denmark (%)	15.3	8.47	0.26
France (%)	54.2	78	**0.007**
Current smoker (%)	7.14	12.7	0.33
Past smoker (%)	51.8	45.5	0.51
Never smoked (%)	30.4	29.1	0.89
Anti-hypertensive treatment (%)	84.7	78	0.35
Number of anti-hypertensive treatments	2	2	0.39
Anti-diabetic treatment (%)	100	78	0.003
Number of anti-diabetic treatments	3	2	**1.80E-07**
Anti-cholesterol treatment (%)	61	52.5	0.36
Number of anti-cholesterol treatments	1	1	0.41
Anti-thrombotic treatment (%)	1.69	1.69	1
Metformin intake (%)	76.3	71.2	0.53
AT2 Receptor Blocker intake (%)	37.3	45.8	0.35
ACE inhibitor intake (%)	30.5	30.5	1
Statin intake (%)	55.9	45.8	0.27

Supplemental Table 6. Characteristics of MetaCardis patients with T2D prescribed Glucagon-like peptide 1 Receptor agonists (GLP-1RAs) vs matched controls. MetaCardis subjects wth diabetes prescribed new generation anti-diabetic drugs (N=59) were propensity-score matched according to

age, sex, disease severity and hyertension status. Estimated Glomerular Filtration Rate (eGFR), Glycated Haemoglobin (HbA1c), Homeostatic Model Assessment for Insulin Resistance (HOMA.IR), Center of recruitment (Germany, Denmark, France). All numeric values are median. Pairwise statistical significance was determined with the two-sided Wilcoxon rank sum tests. *P<0.05.

The MetaCardis Consortium

Petros Andrikopoulos, Judith Aron-Wisnewsky, Rima Chakaroun, Antonis Myridakis, Sofia K. Forslund, Trine Nielsen, Solia Adriouch, Bridget Holmes, Julien Chilloux, Sara Vieira-Silva, Gwen Falony, Joe-Elie Salem, Fabrizio Andreelli, Eugeni Belda, Kanta Chechi, Emmanuelle Le Chatelier, Michael T. Olanipekun, Lesley Hoyles, Renato Alves, Gerard Helft, Richard Isnard, Lars Køber, Luis Pedro Coelho, Christine Rouault, Dominique Gauguier, Jens Peter Gøtze, Edi Prifti, Rohia Alili, Ehm Astrid Andersson Galijatovic, Olivier Barthelemy, Jean-Philippe Bastard, Jean-Paul Batisse, Bel-Lassen Pierre, Magalie Berland, Randa Bittar, Hervé Blottière, Frederic Bosquet, Rachid Boubrit, Olivier Bourron, Mickael Camus, Cecile Ciangura, Jean-Philippe Collet, Arne Dietrich, Morad Djebbar, Angélique Doré, Line Engelbrechtsen, Leopold Fezeu, Sebastien Fromentin, Nicolas Pons, Marianne Graine, Caroline Grünemann, Agnes Hartemann, Bolette Hartmann, Malene Hornbak, Sophie Jaqueminet, Niklas Rye Jørgensen, Hanna Julienne, Johanne Justesen, Judith Kammer, Nikolaj Karup, Ruby Kozlowski, Michael Kuhn, Véronique Lejard, Ivica Letunic, Florence Levenez, Lajos Marko, Laura Martinez-Gili, Robin Massey, Nicolas Maziers, Lucas Moitinho-Silva, Gilles Montalescot, Ana Luisa Neves, Laetitia Pasero Le Pavin, Francoise Pousset, Andrea Rodriguez-Martinez, Sebastien Schmidt, Tatjana Schütz, Lucas Silva, Johanne Silvain, Mathilde Svendstrup, Timothy D Swartz, Thierry Vanduyvenboden, Eric O Verger, Stefanie Walther, Jean-Daniel Zucker, Fredrik Bäckhed, Henrik Vestergaard, Torben Hansen, Jean-Michel Oppert, Matthias Blüher, Jens Nielsen, Jeroen Raes, Peer Bork, Michael Stumvoll, Oluf Pedersen, S. Dusko Ehrlich, Karine Clément, Marc-Emmanuel Dumas.
