# Supplementary material for: Evidence of a causal and modifiable relationship between kidney function and circulating trimethylamine N-oxide
Source: Nat Commun. 2023 Sep 20;14:5843. doi: 10.1038/s41467-023-39824-4 (PMC10511707; doi:10.1038/s41467-023-39824-4)
Supplement: Supplementary file 3 — Description of Additional Supplementary Files [file 41467_2023_39824_MOESM3_ESM.pdf]

Description of Additional Supplementary Files

Supplementary Data 1. List of all variables used in machine learning models. VariablesIDs refer to variable names as displayed in the Source data with DisplayNames the corresponding features. Variable_Groups describe in which feature category each variable was assigned to for the analyses of the study.
