# Supplementary material for: Evidence of a causal and modifiable relationship between kidney function and circulating trimethylamine N-oxide
Source: Nat Commun. 2023 Sep 20;14:5843. doi: 10.1038/s41467-023-39824-4 (PMC10511707; doi:10.1038/s41467-023-39824-4)
Supplement: Supplementary file 5 — Reporting Summary [file 41467_2023_39824_MOESM5_ESM.pdf]

nature portfolio

Corresponding author(s): Marc-Emmanuel Dumas
Karine Clément
Petros Andrikopoulos

Last updated by author(s): May 02, 2023

Reporting Summary

Nature Portfolio wishes to improve the reproducibility of the work that we publish. This form provides structure for consistency and transparency in reporting. For further information on Nature Portfolio policies, see our Editorial Policies and the Editorial Policy Checklist.

Statistics

For all statistical analyses, confirm that the following items are present in the figure legend, table legend, main text, or Methods section.

n/a	Confirmed	
☐	☒	The exact sample size (*n*) for each experimental group/condition, given as a discrete number and unit of measurement
☒	☐	A statement on whether measurements were taken from distinct samples or whether the same sample was measured repeatedly
☐	☒	The statistical test(s) used AND whether they are one- or two-sided<br>*Only common tests should be described solely by name; describe more complex techniques in the Methods section.*
☐	☒	A description of all covariates tested
☐	☒	A description of any assumptions or corrections, such as tests of normality and adjustment for multiple comparisons
☐	☒	A full description of the statistical parameters including central tendency (e.g. means) or other basic estimates (e.g. regression coefficient) AND variation (e.g. standard deviation) or associated estimates of uncertainty (e.g. confidence intervals)
☐	☒	For null hypothesis testing, the test statistic (e.g. *F*, *t*, *r*) with confidence intervals, effect sizes, degrees of freedom and *P* value noted<br>*Give P values as exact values whenever suitable.*
☒	☐	For Bayesian analysis, information on the choice of priors and Markov chain Monte Carlo settings
☒	☐	For hierarchical and complex designs, identification of the appropriate level for tests and full reporting of outcomes
☐	☒	Estimates of effect sizes (e.g. Cohen's *d*, Pearson's *r*), indicating how they were calculated

Our web collection on statistics for biologists contains articles on many of the points above.

Software and code

Policy information about availability of computer code

Data collection	See Methods section for details; METEOR v3.2 (https://forgemia.inra.fr/metagenopolis/meteor), Alientrimmer:v0.4.0, Bowtie2 v2.3.4, MetaOMineR (momr, v1.31), Omixer-RPM (v1.0) were each used to process microbiome data. MassLynxTM (Waters corporation; Version 4.2) software was used for UPLC-MS/MS data acquisition and analysis. 1H-NMR absolute quantifications were derived using the "In Vitro Diagnostics for research" (IVDr) algorithm (Bruker; v1.1). Image analyses of western blot films and immunohistochemistry slides was conducted with ImageJ (NIH; v1.46r)
Data analysis	Analysis was conducted using the R (v4.03) statistical language as described in the Methods. No custom software was used in this project.

For manuscripts utilizing custom algorithms or software that are central to the research but not yet described in published literature, software must be made available to editors and reviewers. We strongly encourage code deposition in a community repository (e.g. GitHub). See the Nature Portfolio guidelines for submitting code & software for further information.

Data

Policy information about availability of data

All manuscripts must include a data availability statement. This statement should provide the following information, where applicable:

- Accession codes, unique identifiers, or web links for publicly available datasets
- A description of any restrictions on data availability
- For clinical datasets or third party data, please ensure that the statement adheres to our policy

The Raw shotgun sequencing data that support the findings of this study have been deposited in the European Nucleotide Archive under accession codes

“PRJEB37249 [https://www.ebi.ac.uk/ena/browser/view/PRJEB37249]”, “PRJEB38742 [https://www.ebi.ac.uk/ena/browser/view/PRJEB38742]”, “PRJEB41311 [https://www.ebi.ac.uk/ena/browser/view/PRJEB41311]” and “PRJEB46098[https://www.ebi.ac.uk/ena/browser/view/PRJEB46098]”. The Serum GC-MS and isotopically quantified serum metabolites (UPLC–MS/MS) that have been used in this study are deposited in MassIVE with accession numbers “MSV000088042 [https://doi.org/doi:10.25345/C5CV76]” and “MSV000088043[https://doi.org/doi:10.25345/C58246]”, respectively.

In adherence to EU and national privacy laws, patient phenotypic data will be made available to bona fide researchers upon request, from the corresponding authors, following approval by the relevant EU and national Data Protection Agencies. Corresponding authors will advise interested researchers about the correct procedure to make such applications within 5 working days but will not share any costs or make the application on their behalf. Source data are provided with this paper.

Human research participants

Policy information about studies involving human research participants and Sex and Gender in Research.

Reporting on sex and gender	Our study comprises 864 Female and 890 Male individuals. Therefore, our study findings apply to both sexes. For French and Danish patients biological sex was assigned at recruitment by a physician. For German patients biological sex was taken from the patient's insurance card and was assigned at birth.
Population characteristics	Patients were subclassified in three groups: BMI-spectrum patients (BMIS; N=837), encompassing MetaCardis participants presenting with metabolic syndrome-related risk factors or conditions (hypertension, obesity and metabolic syndrome) and patients diagnosed with type-2 diabetes (T2D; N=561) or ischaemic heart disease (IHD; N=356). The IHD group comprised patients with Acute (<15days) Coronary Syndrome (ACS; N=106), Chronic IHD (CIHD; N=157) with normal Left Ventricular Ejection Fraction (LVEF) determined by echocardiography and Heart Failure patients (HF; N=93, LVEF<45%). Subgroup characteristics are appended in Supplemental Table 1.
Recruitment	MetaCardis is a cross-sectional study that recruited individuals at increasing stages of dysmetabolism and IHD severity (ranging from metabolically healthy, metabolic syndrome and/or obesity, T2D, IHD), aged 18–75 years old and recruited from Denmark, France and Germany between 2013 and 2015. Patients under care of the participating hospitals were invited to enroll where fulfilling the criteria of the study and healthy controls were recruited via public advertisement. Study participants provided written informed consent and the study was undertaken according to Helsinki Declaration-II.
Ethics oversight	Ethical approval was obtained from the Ethics Committee CPP Ile-de France, the Ethical Committees of the Capital Region of Denmark (H-3-2013-145) and Ethics Committee at the Medical Faculty at the University of Leipzig (047-13-28012013)

Note that full information on the approval of the study protocol must also be provided in the manuscript.

Field-specific reporting

Please select the one below that is the best fit for your research. If you are not sure, read the appropriate sections before making your selection.

[x] Life sciences [ ] Behavioural & social sciences [ ] Ecological, evolutionary & environmental sciences

For a reference copy of the document with all sections, see nature.com/documents/nr-reporting-summary-flat.pdf

Life sciences study design

All studies must disclose on these points even when the disclosure is negative.

Sample size	No prior power calculation was carried out but sample size is comparable to that of the MetaCoronary study (Fromentin S, et al. Microbiome and metabolome features of the cardiometabolic disease spectrum. Nat Med 2022;28:303-314.doi:10.1038/s41591-022-01688-4), which was adequately powered.
Data exclusions	No subjects for whom data was available were excluded during analysis.
Replication	We replicate the main finding of our study suggesting direct effect of TMAO on the kidney using primary human fibroblasts and the gold-standard animal model of kidney fibrosis in the absence of other co-morbidities. For the cellular studies there were 3 independent biological repeats. The animal study was conducted once with 6 animals per experimental group. All experimental groups were treated concurrently.<br><br>The reno-protective properties of Glucagon-like peptide-1 receptor agonists (GLP-1RAs), have been reported in large trials (Shaman AM, et al. Effect of the Glucagon-Like Peptide-1 Receptor Agonists Semaglutide and Liraglutide on Kidney Outcomes in Patients With Type 2 Diabetes: Pooled Analysis of SUSTAIN 6 and LEADER. Circulation 2022;145:575-585), referenced in our manuscript and therefore we do not attempt to replicate this here.
Randomization	In the human part of the study no intervention or experiment was made, only observation. Therefore, since there is no intervention to randomize, randomization is neither applicable nor relevant. In the animal study, mice were randomly assigned to experimental groups. Moreover, animals were purchased at the same time from the same supplier and housed in the same room. Dietary intervention was initiated after a 10-day acclimatization period and all experimental groups were treated concurrently. Additionally, surgical procedures and subsequent culling were performed on the same day at a random order.

Blinding	Analysts (data managers, statisticians, bioinformaticicans) were blinded by having access only to pseudonymized data, and performed no manual analyses - all statistics and visualization were undertaken using computer software. As such, no analysts awareness of any group allocation (diagnosis) affected outcomes of any statistical analysis, and only results of such analysis in aggregate were used to draw conclusions and for interpretations of results. In this sense analysis is as blinded as is at all possible in an -omics biomarker study, and in line with standards of the field.<br><br>The experimentalists in the animal and cell-based part of our study were not blinded. This is because cellular experiments and phenotypic analyses of the animals were conducted by one person and therefore blinding was not practical.

Reporting for specific materials, systems and methods

We require information from authors about some types of materials, experimental systems and methods used in many studies. Here, indicate whether each material, system or method listed is relevant to your study. If you are not sure if a list item applies to your research, read the appropriate section before selecting a response.

Materials & experimental systems

n/a	Involved in the study
☐	☒ Antibodies
☐	☒ Eukaryotic cell lines
☒	☐ Palaeontology and archaeology
☐	☒ Animals and other organisms
☐	☒ Clinical data
☒	☐ Dual use research of concern

Methods

n/a	Involved in the study
☒	☐ ChIP-seq
☒	☐ Flow cytometry
☒	☐ MRI-based neuroimaging

Antibodies

Antibodies used	The following antibodies were used in the present study with dilutions in parentheses: From Cell Signaling Technology, Phospho-p44/42 MAPK (Erk1/2) (Thr202/Tyr204) Antibody (1:3000) #9101, p44/42 MAPK (Erk1//2) Antibody (1:5000) #9102, Phospho-Smad3 (Ser423/425) (C25A9) Rabbit mAb (1:1000) #9520, Phospho-p70 S6 Kinase (Thr389) Antibody (1:1000) #9205, Phospho-S6 Ribosomal Protein (Ser235/236) Antibody (1:1000) #2211, Phospho-4E-BP1 (Ser65) Antibody (1:1000) #9451, Vimentin (R28) antibody (1:1000) #3932. From Sigma-Aldrich, monoclonal anti-actin, α-Smooth Muscle (clone 1A4) A2547 (1:10000), monoclonal Anti-α-Tubulin antibody (clone DM1A; 1:10000) T9026, anti-β-actin antibody (clone AC-74, 1:10000) A2228.<br><br>For immunohistochemistry, the Anti-F4/F80 (Cl:A3-1) antibody (1:50) from Biorad (MCA497) was used.
Validation	All the antibodies were sourced commercially and extensively reported in the literature and validated by the vendors. Therefore, no specific validation was performed. Below the vendor websites for each antibody where its validation is described. Where available citations in the literature are also given for each antibody.<br><br>Phospho-p44/42 MAPK (Erk1/2) (Thr202/Tyr204) Antibody #9101,<br>https://www.cellsignal.com/products/primary-antibodies/phospho-p44-42-mapk-erk1-2-thr202-tyr204-antibody/9101?site-search-type=Products&N=4294956287&Ntt=%239101&fromPage=plp&_requestid=878708. (7878 citations)<br><br>p44/42 MAPK (Erk1//2) Antibody (1:5000) #9102,<br>https://www.cellsignal.com/products/primary-antibodies/p44-42-mapk-erk1-2-antibody/9102?site-search-type=Products&N=4294956287&Ntt=%239102&fromPage=plp&_requestid=878862. (15792 citations)<br><br>Phospho-Smad3 (Ser423/425) (C25A9) Rabbit mAb (1:1000) #9520,<br>https://www.cellsignal.com/products/primary-antibodies/phospho-smad3-ser423-425-c25a9-rabbit-mab/9520?site-search-type=Products&N=4294956287&Ntt=%239520&fromPage=plp&_requestid=879127. (770 citations)<br><br>Phospho-p70 S6 Kinase (Thr389) Antibody (1:1000) #9205,<br>https://www.cellsignal.com/products/primary-antibodies/phospho-p70-s6-kinase-thr389-antibody/9205?site-search-type=Products&N=4294956287&Ntt=%239205&fromPage=plp&_requestid=879237. (1698 citations)<br><br>Phospho-S6 Ribosomal Protein (Ser235/236) Antibody (1:1000) #2211,<br>https://www.cellsignal.com/products/primary-antibodies/phospho-s6-ribosomal-protein-ser235-236-antibody/2211?site-search-type=Products&N=4294956287&Ntt=%232211&fromPage=plp&_requestid=879346. (1405 citations)<br><br>Phospho-4E-BP1 (Ser65) Antibody (1:1000) #9451,<br>https://www.cellsignal.com/products/primary-antibodies/phospho-4e-bp1-ser65-antibody/9451?site-search-type=Products&N=4294956287&Ntt=%239451&fromPage=plp&_requestid=879460. (507 citations)<br><br>Vimentin (R28) antibody (1:1000) #3932.<br>https://www.cellsignal.com/products/primary-antibodies/vimentin-r28-antibody/3932?site-search-

type=Products&N=4294956287&Ntt=%233932&fromPage=plp&_requestid=879975. (225 citations)

Monoclonal anti-actin, α-Smooth Muscle (clone 1A4) A2547 (1:10000), https://www.sigmaaldrich.com/GB/en/product/sigma/a2547

monoclonal Anti-α-Tubulin antibody (clone DM1A; 1:10000) T9026, https://www.sigmaaldrich.com/GB/en/product/sigma/t9026

anti-β-actin antibody (clone AC-74, 1:10000) A2228. https://www.sigmaaldrich.com/GB/en/product/sigma/a2228

Anti-F4/F80 (Cl:A3-1) antibody (1:50) from Biorad (MCA497) https://www.bio-rad-antibodies.com/monoclonal/mouse-f4-80-antibody-cl-a3-1-mca497.html?f=purified (228 citations)

Eukaryotic cell lines

Policy information about cell lines and Sex and Gender in Research

Cell line source(s)	Primary human renal fibroblasts were obtained from DV Biologics (AU009-F).
Authentication	The cells were of commercial source, had the characteristic enlogated fibroblast-like appearance under the microscope and upon experimentation expressed fibroblast markers and responded to TGF-beta-1. Therefore, no specific tests were carried to verify their fibroblast origin.
Mycoplasma contamination	Cells were tested for mycoplasma at culture initiation on arrival.
Commonly misidentified lines (See ICLAC register)	N/A

Animals and other research organisms

Policy information about studies involving animals; ARRIVE guidelines recommended for reporting animal research, and Sex and Gender in Research

Laboratory animals	C57BL/6J males, 6-8weeks of age at the start of the experiment. Food and water were available ad libitum and mice were held at 12/12h light/dark cycle at 20-23oC and 40-60% humidity in standard individually ventilated cages (5-6 animals in each cage).
Wild animals	No wild animals were used in this study
Reporting on sex	We only used male mice, as common practice with this model. Therefore, the findings of the animal study apply only to male animals. However, since we did not observe a strong effect for sex on TMAO levels and responses in the human part of our study, it is reasonable to assume that the effects of TMAO on renal fibrosis we identified in male mice would also apply to females as well.
Field-collected samples	No field-collected samples were used in this study.
Ethics oversight	United Kingdom Home Office, Project License 70/8356.

Note that full information on the approval of the study protocol must also be provided in the manuscript.

Clinical data

Policy information about clinical studies
All manuscripts should comply with the ICMJEguidelines for publication of clinical research and a completedCONSORT checklist must be included with all submissions.

Clinical trial registration	The study protocol was registered at clinicaltrial.gov (NCT02059538).
Study protocol	Available from the study promoter: Assistance Publique-Hôpitaux de Paris (AP-HP).
Data collection	MetaCardis is a cross-sectional study that recruited individuals at increasing stages of dysmetabolism and IHD severity (ranging from metabolically healthy, metabolic syndrome and/or obesity, T2D, IHD), aged 18–75 years old and recruited from Denmark, France and Germany between 2013 and 2015. Patients under care of the participating hospitals meeting the inclusion criteria of the study were invited to enroll. Healthy controls were recruited via public advertisement. Study participants provided written informed consent and the study was undertaken according to Helsinki Declaration-II.
Outcomes	MetaCardis is an observational study, therefore, no outcomes were measured.
